# Defining a successful total knee arthroplasty: a systematic review of metrics of clinically important changes

**DOI:** 10.1186/s42836-023-00178-3

**Published:** 2023-05-18

**Authors:** Zodina A. Beiene, Kira K. Tanghe, Cynthia A. Kahlenberg, Alexander S. McLawhorn, Catherine H. MacLean, Elizabeth B. Gausden

**Affiliations:** 1 Department of Anesthesiology and Critical Care, Johns Hopkins Hospital, 1800 Orleans Street, Baltimore, MD 21287 USA; 2 Albert Einstein Medical College, Bronx, NY 10461 USA; 3 Department of Orthopedic Surgery, Hospital for Special Surgery, New York, NY 10021 USA; 4 Center for the Advancement of Value in Musculoskeletal Care, Hospital for Special Surgery, New York, NY 10021 USA; 5 Department of Medicine, Weill Cornell Medical College, New York, NY 10021 USA

**Keywords:** Total knee arthroplasty, Total knee replacement, Minimal clinically important difference, Patient acceptable symptom state, Patient-reported outcome measure

## Abstract

**Background:**

Despite the increasing use of patient-reported outcome measures (PROMs), the methodology used to evaluate clinically significant postoperative outcomes after total knee arthroplasty (TKA) is variable. The review aimed to survey studies with identified PROM-based metrics of clinical efficacy and the assessment procedures after TKA.

**Methods:**

The MEDLINE database was queried from 2008–2020. Inclusion criteria were: full texts, English language, primary TKA with minimum one-year follow-up, use of metrics for assessing clinical outcomes with PROMs, and primary derivations of metrics. The following PROM-based metrics were identified: minimal clinically important difference (MCID), minimum detectable change (MDC), patient acceptable symptom state (PASS), and substantial clinical benefit (SCB). Study design, PROM value data, and methods of derivation for metrics were recorded.

**Results:**

We identified 18 studies (including 46,173 patients) that met the inclusion criteria. Across these studies, 10 different PROMs were employed, and MCID was derived in 15 studies (83%). The MCID was calculated using anchor-based techniques in nine studies (50%) and distribution techniques in eight studies (44%). PASS values were presented in two studies (11%) and SCB in one study (6%) using an anchor-based method; MDC was derived in four studies (22%) using the distribution method.

**Conclusion:**

There is variability in the TKA literature with respect to the definition and derivation of measurements of clinically significant outcomes. Standardization of these values may have implications for optimal case selection and PROM-based quality measurement, ultimately improving patient satisfaction and outcomes.

**Supplementary Information:**

The online version contains supplementary material available at 10.1186/s42836-023-00178-3.

## Introduction

Patient-reported outcome measures (PROMs) can be used to assess the efficacy of total knee arthroplasty (TKA), an elective procedure that patients undergo to reduce their knee pain and improve function. They are a directly reported assessment by patients of their state at a specific time point [1, 2]. Therefore, they are valuable to clinicians and researchers in determining a change in a patient’s perceived state. However, there are many challenges to overcome to consistently and precisely use PROMs to assess clinical efficacy.

Despite the increased use of PROMs, there is variability in the methods used to evaluate clinically significant change and subsequent interpretation of results. Metrics of clinically important differences allow clinicians to apply significant results to their patients. The minimal clinically important difference (MCID) is one well-known metric established to relate changes in instrument scores to clinically important outcomes. Historically, it has been defined as "the smallest difference in score in the domain of interest which patients perceive as beneficial" [3, 4] and would likely repeat intervention if presented with the choice again. Values exceeding this benchmark indicate a clinically important change. MCID is the most commonly reported measure, however variably derived and reported.

Currently used measures of clinical significance conceptually similar to MCID also include clinically important difference (CID) [5], minimal clinically important improvement (MCII) [6], minimal detectable change (MDC), the minimal important difference (MID), and minimal important change (MIC) [7]. Rather than represent a floor value for clinical improvement, substantial clinical benefit (SCB) is defined as a threshold indicating "optimal clinical benefit" [8]. Similarly, patient acceptable symptom state (PASS) is a threshold measure above which acceptable satisfaction has been achieved [9]. This study aimed to assess the use of metrics of clinically important change and methods of derivation when using PROMs in TKA research and clinical practice.

## Materials and methods

### Search strategy

The MEDLINE database was queried from 1 January 2008 to 8 October 2020. The search strategy included a combination of text words and medical subject headings, including clinically significant change and total knee and hip (THA) arthroplasty. We searched the MEDLINE database for the following phrases after TKA: "smallest detectable difference (SDD)," "minimal detectable change (MDC)," "minimal clinically important change (MCIC)," "minimal clinically important improvement (MCII)," "minimal clinically important difference (MCID)," "clinically important difference (CID)," "substantial clinical benefit (SCB)," "patient acceptable symptom state (PASS)," or "outcome assessment (health care)/statistics and numerical data." These phrases were combined with the following terms: "total joint replacement," "total joint arthroplasty," "total knee arthroplasty," "total knee replacement," "arthroplasty, replacement, knee," "arthroplasty," or "arthroplasty, replacement."

Studies were included if PROM-based quantitative metrics for assessment of clinically significant improvement were used and primarily derived. Additional inclusion criteria were: full text, English language, and a minimum of one-year follow-up postoperatively. Studies were limited to randomized controlled trials, prospective and retrospective cohorts, and case–control studies. Study design, PROM data, and methods of derivation for metrics of clinically significant change were recorded. Selected THA studies that satisfied inclusion criteria were analyzed and later discussed in a separate corollary study.

### Study selection

**Fig. 1:**
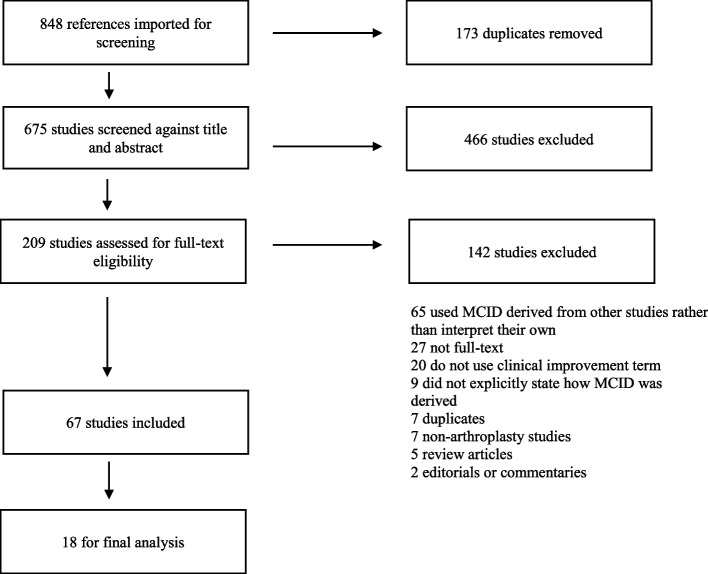
A total of 67 studies were included after full-text assessment, and 18 TKA studies met follow-up (≥ 1 year) and PROM language (English) criteria

We used Covidence, a systematic review management platform, to screen and extract studies according to Preferred Reporting Items for Systematic Review and Meta-Analysis (PRISMA) guidelines [10]. Duplicates were identified and eliminated by the screening algorithm. Four reviewers independently screened the titles and abstracts (C.A.K., E.B.G., K.K.T., and Z.A.B.). Exclusion criteria were as follows: non-English language, non-Human subjects, the absence of aforementioned keywords for assessing clinical improvement after unilateral or bilateral TKA, the absence of outcomes of the studies, non-full text, non-total knee arthroplasty interventions, and a clinical improvement term not primarily calculated but rather reported by referencing previous studies. The full TKA articles were then evaluated independently by three reviewers for eligibility (E.B.G., K.K.T., and Z.A.B.). There was at least one senior resident screening at each stage (C.A.K., E.B.G.). Discrepancies between reviewers were resolved by discussion. Between two reviewers (C.A.K., E.B.G.), there were 22 discrepancies (Cohen’s Kappa 0.89, 95% proportion agreement). There were 32 discrepancies between the two other reviewers (Z.A.B., K.K.T.; Cohen’s Kappa 0.66, 83% proportion agreement). These discrepancies may, in part, be attributed to the level of training and years of clinical experience. Sixty-seven studies were included (See Fig. 1). From there, studies using non-English-based PROMs with less than one year of follow-up were excluded. Eighteen TKA studies were included for final analysis (See Table 1).

**Table 1:** Study demographics arranged by design, PROM, change score, method of derivation, and follow-up

First author	Year	Study design	Total patients included^a^	PROM(s)	Change score(s)	Method of calculation	Follow-up (year)
Berliner [11]	2017	3	562	KOOS, SF-12	MCID	Distribution	1
Blevins [12]	2019	3	228	KOOS, SF-12	MCID	Distribution	2
Busija [13]	2008	4	379 (105)	SF-36	MDC	Distribution	0.5, 1; 5
Chesworth [5]	2008	2	3,657 (1,578)	WOMAC	CID	Anchor	1
Clement [14]	2013	2	505	OKS, SF-12	MCID	Anchor	1
Clement [15]	2018	3	2,589	WOMAC	MCID, MDC, MIC	Both^b^	1
Clement [16]	2019	3	2,589	SF-12	MCID, MDC, MIC	Both^b^	1
Connelly [17]	2019	3	383; 301	KOOS, EQ-5D/VAS, NRS	PASS	Anchor	1; 3
Fontana [18]	2019	3	13,719 (6,480)	SF-36	MCID	Distribution	2
Goodman [19]	2020	3	10,622 (4,641)	KOOS	MCID	Anchor	2
Harris [20]	2021	2	637	KOOS	MCID	Anchor	1
Kagan [21]	2018	2	91	PROMIS	MCID	Distribution	0.13; 0.25; 0.5; 1
Kuo [22]	2020	2	1,088 (587)	KOOS	MCID	Both	1
Lawrie [23]	2020	3	172	PROMIS	MCID	Distribution	1
Lyman [24]	2018	3	4,953 (2,630)	KOOS	MCID, MDC, SCB	Both^c^	2
Maxwell [25]	2013	2	271	WOMAC	PASS	Anchor	2.5; 5
Razak [26]	2016	3	3,328	OKS, SF-36	MCID	Distribution	5
Sayers [6]	2017	2	400 (190)	ICOAP	MCID, MID	Both^d^	0.25; 0.5; 1

Study design: 1 = Randomized control trial, 2 = prospective cohort, 3 = retrospective cohort, 4 = case–control

*MCID* minimal clinically important difference, *MDC* minimum detectable change, *CID* clinically important difference, *MIC* minimal important change, *PASS* patient acceptable symptom state, *SCB* substantial clinical benefit. *KOOS* Knee Injury and Osteoarthritis Outcome Score, *SF-12* Short Form-12, *SF-36* Short Form-36, *WOMAC* Western Ontario McMaster University osteoarthritis index, *OKS* Oxford Knee Score, *NRS* Numeric Pain Rating Scale, *PROMIS* Patient-Reported Outcomes Measurement Information System, *ICOAP* Intermittent and Constant Osteoarthritis Pain questionnaire, *EQ-5D-3L/VAS* EuroQoL 5-dimension 3-level and visual analog scale

^a^Parenthesis indicates cohort of TKA patients specifically

^b^MCID derived by anchor, MDC by distribution method, and MIC by anchor method

^c^MCID derived by both methods, MDC by distribution, and SCB by anchor method

^d^MCID derived by anchor, MID by distribution method

### Outcome measures

PROM data and values of clinical improvement, including the use of preoperative thresholds for achieving clinically significant change, were extracted. Methods of calculation for these values were identified and stratified according to PROM(s) used. The use of comparative groups and special patient populations was also observed. Any predictors of outcome were recorded.

Globally, Knee Injury and Osteoarthritis Outcome Score (KOOS) (on a 0–100 point scale) contains domains of pain, symptoms, function in daily living, function in sport and recreation, and quality of life, with a higher score indicating an improved status [27, 28]. Western Ontario McMaster University osteoarthritis index (WOMAC) (ranging from 0–96 points) contains pain, stiffness, and function domains, with a higher score indicating a worse outcome [29]. Short Form-12 (SF-12) (0–200 points) is a generic health status scale that includes a physical and mental component score, with a higher score indicating a better outcome [30]. Short Form-36 (SF-36) score (0–100 points) is a generic quality of life measure with eight domains including pain and physical functioning, with a higher score indicating better health [31, 32]. Oxford Knee Score (OKS) (12–60 points) contains 12 components assessing pain and functional limitations, with a higher score indicating a worse outcome [33, 34].

Additionally, the Patient-Reported Outcomes Measurement Information System (PROMIS) assesses physical function and includes physical and mental health domains, with low scores representing low physical function. PROMIS scores are normalized to the general population using a T-score [35]. Intermittent and Constant Osteoarthritis Pain (ICOAP) (0–100 points) assesses constant and intermittent pain, with a higher score indicating a worse outcome [36]. EuroQoL 5-dimension 3-level (EQ-5D-3L) is a health-related quality of life measure with five domains (mobility, self-care, usual activities, pain and discomfort, and anxiety and depression), each rated as no, some, or extreme problems. The Visual Analog Scale (VAS) (0–100 points) component is an overall measure of health, with a higher score indicating better health [37]. The Numeric Rating Scale (NRS) (0–20 points) is a 21-point pain scale, with higher scores indicating severe postoperative pain [38].

### Methods of calculation in the literature

Three approaches were used in the literature to determine values marking clinical significance: anchor-based, distribution, and expert or consensus methods. The studies examined in this review primarily employed anchor and distribution methods. The former method applies a subjective clinical question to PROM change scores and the latter is a statistical measurement that compares PROM change scores to errors of measurement (See Supplementary). Anchor-based values were obtained using simple linear regression analysis [14] or receiver operating characteristic curves (ROC) at maximum sensitivity and specificity [5, 15, 17, 22, 24] to identify PROM change scores that distinguish between those who are “better” from the unchanged.

## Results

We identified 18 studies (involving a total of 46,173 patients) that met the inclusion criteria (See Table 1). Among these, 10 different PROMs were studied: KOOS (7 studies) [11, 12, 17, 19, 20, 22, 24], WOMAC (3 studies) [5, 15, 25], SF-12 (4 studies) [11, 12, 14, 16], SF-36 (3 studies) [13, 18, 26], OKS (2 studies) [14, 26], PROMIS (2 studies) [21, 23], ICOAP (1 study) [6], EQ-5D-3L/VAS (1 study) [17], and NRS (1 study) [17].

### MCID

MCID or CID was derived in 15 studies for the following PROMs: KOOS/KOOS, JR (range, 6–25) [11, 12, 19, 20, 22, 24], WOMAC (range, 8–36) [5, 15], SF-12 (range, 2–5) [11, 12, 14, 16], and SF-36 (range, 5–10) [18, 26], OKS (range, 4–5) [14, 26], and PROMIS physical function computerized adaptive test (CAT) (range, 3–5) [21, 23], and ICOAP (chronic pain, 24) [6] (See Table 2). The MCID was calculated using anchor-based techniques in nine studies (50%) [5, 6, 14–16, 19, 20, 22, 24] and distribution techniques in eight studies (44%) [11, 12, 18, 21–24, 26] as their primary mode of calculation. Two studies used both techniques (11%) [22, 24].

**Table 2:** MCID, PASS and MDC ranges by PROM and method

PROM	MCID range		PASS range		MDC range
	Distribution	Anchor	Distribution	Anchor	Distribution
KOOS	6.0–15.8^1^	7.0–25.0^2^		66.0–84.5, 66.0–84.5, 63.0–84.0 (1 year); 66.0–87.5, 66.0–90.5, 64.5–87.0 (3 year)	
KOOS, JR	6.0–8.7^3^	14.0–20.8^4^			7.0–11.0^1^
WOMAC		8.0–36.0^5^	Threshold 22^a^		11.0–27.0^2^
OKS	5.0^6^	4.3–5.0^7^			
PROMIS	3.3–5.0^8^				
SF-12	5.0–5.4^9^	1.5–4.8^10^			8.9–13.8^3^
SF-36	5.0–10.0^11^				35.0–94.0 (individual); 5.0–14.0 (group)^4^
ICOAP		23.7^12^			
EQ-VAS				83.0, 71.0, 70.0 (1 year); 90.5, 74.5, 77.5 (3 year)	
EQ-5D-3L				0.80, 0.75, 0.80 (1 year); 0.80, 0.79, 0.80 (3 year)	
NRS				1.8, 2.2, 1.5 (1 year); 1.8, 1.2, 1.0 (3 year)	

MCID ranges by PROM using both methods: 1. Berliner 2017, Blevins 2019, Kuo 2020, Lyman 2018; 2. Goodman 2020, Harris 2021, Kuo 2020, Lyman 2018; 3. Kuo 2020, Lyman 2018; 4. Harris 2021, Kuo 2020, Lyman 2018; 5. Chesworth 2008 (CID), Clement 2018; 6. Razak 2016; 7. Clement 2013; 8. Kagan 2018, Lawrie 2020; 9. Berliner 2017, Blevins 2019; 10. Clement 2013 (includes pain and function), Clement 2019; 11. Razak 2016 (PCS), Fontana 2019; 12. Sayers 2017 (chronic pain). PASS ranges by PROM using primarily anchor-based methods: Connelly 2019, reported by 80% specificity, Youden index, and the 75th percentile

MDC ranges by PROM using only distribution methods: 1. Lyman 2018 (MDC-80, 90, 95); 2. Clement 2018 (MDC-95); 3. Clement 2019 (MDC-90); 4. Busija 2008 (MDC-95). MDCgroup = MDCindividual/)/√n.; Where MDC = z-score $\times$ SEM $\times$ √2. SEM = SD $\times$ √(1–reliability). *SEM* standard error measurement, *SD* standard deviation

^a^Maxwell 2013—back-calculated

### KOOS

Four of the seven studies that used the KOOS scale [19, 20, 22, 24] had anchor-based questions to determine MCID: (1) change defined by the response "a little improvement" on the quality of life (QOL) question, which was further queried with how total joint replacement changed the QOL [19], (2) the Self-Administered Patient Satisfaction Scale (SAPS), an anchor questionnaire, assessing satisfaction with results of surgery, improvement of pain, improvement in ability to do home or yard work, and improvement in ability to do recreational activities [20, 22], and (3) "How much did knee surgery improve the quality of your life?" on the Hospital for Special Surgery (HSS) satisfaction survey [24]. For distribution techniques, four studies used one-half the standard deviation (SD) of baseline scores and change scores from baseline to follow-up [11, 12, 22, 24].

For some PROMs, MCID values varied by derivation method. KOOS, JR specifically ranged from 6–9 by distribution [22, 24] and 14–21 by anchor-based methods [20, 22, 24]. KOOS, JR 21.0, 17.5, 14.0 corresponded to anchor questions (2) and (3) as mentioned above [20, 22, 24] (See Table 2). Goodman et al. reported KOOS pain and function subscales anchored on "a little improvement" (question 1 as aforementioned) as 21.0 and 14.2, respectively [19]. Blevins et al. reported 10.3 and 12.0 for KOOS pain and symptom subscales by distribution method [12].

### WOMAC

For two of the three studies that used the WOMAC index [5, 15], anchor-based questions were: (1) "Whether compared to when they went on the waitlist for surgery, were they better, worse, or the same?" and "Knowing what your hip or knee replacement surgery did for you, if you could go back in time, would you still have undergone this surgery?” and (2) "How much did the knee replacement surgery improve the quality of your life?" MCID values anchored on "a good deal better" for WOMAC pain and function were 36 and 33, respectively. Values anchored on "willing to have index surgery again" for WOMAC pain and function were 31 and 26, respectively [5]. No studies used distribution-based techniques for the WOMAC (See Table 2).

### SF-12

For two of the four studies that used the SF-12 scale [14, 16], anchors included: (1) "How well did the surgery relieve pain in your affected joint?" and "How well did the surgery increase your ability to perform regular activities?" and (2) "How much did the knee replacement surgery improve the quality of your life?" Values calculated via the distribution method used one-half the SD of change scores [11, 12]. Physical component scores (PCS) were 1.8 vs. 5.0 and mental component scores (MCS) were 1.5 vs. 5.4 for anchor vs. distribution methods (See Table 2).

### SF-36, PROMIS, OKS, and ICOAP

For SF-36 and PROMIS scales, all four studies used only distribution methods to obtain the MCID, which was one-half the SD [18, 21, 23, 26]. For two studies that administered the OKS scale, MCID was calculated by the distribution method, which again was one-half the SD [26] and anchor method [14], respectively. For the one study that utilized ICOAP, MCID was derived via an anchor approach [6] (See Table 2). Distribution-obtained MID in the same study was 11.8 [6].

### PASS

PASS values were presented in two studies [17, 25] for the following PROMs: KOOS (range, 66–91), EQ-5D-3L (range, 0.75–0.80), EQ-VAS (range, 70–91), and NRS (range, 1–2.2). The anchor question used was "How satisfied are you with the result of your most recent knee treatment?" Three different methods of calculation were used to obtain the above values: 80 percent specificity, Youden index, and the 75th percentile (See Table 2, Supplementary).

### MDC

MDC is defined as the minimum amount of change capturing true clinical change rather than mere variability associated with repeated PROM measurements. Scores above the MDC represent true improvement within a certain degree of confidence according to the chosen confidence interval [39]. MDC values were obtained in four studies [13, 15, 16, 24] using exclusively distribution methods with the standard error of measurement (SEM) and either 80, 90 or 95 percent confidence intervals for the following PROMs: KOOS, WOMAC, SF-12, and SF-36 (See Table 2). Two studies [15, 16] obtained both MDC-95, -90 percentiles and MCID values using distribution and anchor methods, respectively.

### SCB and MIC

SCB was obtained in one study for KOOS, JR (20.0) [24] using an anchor-based ROC approach (See Supplementary). The anchor was the QOL question on the HSS satisfaction survey. The SCB value exceeded both MCID and MDC values for the JR version. MIC was obtained in two studies for WOMAC (range, 13–21) [15] and SF-12 PCS (2.7) [16] using anchor-based ROC curves.

### Preoperative predictors

In all, three studies (18%) used a comparative group [12, 13, 23]. One study had a special patient population (i.e. rheumatoid arthritis) [12]. The most commonly reported predictors of outcome in reaching the MCID or SCB included preoperative PROMs, age, and comorbidities. For example, significant predictors of achieving the MCID for OKS at five years were age (younger age), the Knee Society Knee Score (KSKS) (lower score), and the Knee Society Function Score (KSFS) (lower score) [26]. Preoperative KOOS < 58 and SF-12 PCS < 34 were associated with an increased likelihood of achieving clinically significant improvement after TKA [11]. For one study deriving SCB values, predictors of the outcome included age, gender, body mass index (BMI), American Society of Anesthesiologists class, and the Charlson Comorbidity Index [24].

## Discussion

There is substantial heterogeneity in the arthroplasty literature with regard to the definition, measurement, and reporting of clinically meaningful changes. We found that values of clinical improvement varied according to PROM and method of derivation. Anchor methods were more frequently used for MCID and PASS values and modes of derivation were heterogeneous. Anchor-derived MCID values were greater than distribution-derived ones.

Clinical improvement terms differ subtly by definition and are not necessarily comparable or interchangeable, contributing to the heterogeneity. Terms often used synonymously with MCID, however, are more nuanced in definition, such as applicability to individual or group settings. For example, CID was defined in one study as any change, not exclusively minimum, either positive or negative anchored on "a good deal better" within a patient group. ROC curves were generated to identify CID values at the level of the individual [5]. MIC is generally defined as a change within an individual or group over time. More specifically, it was defined in one study as a change in PROM score relative to baseline for patients who reported meeting the anchor "little improvement" and calculated on the individual level using ROC curves [15]. MID is defined as the minimal important difference when comparing two groups of patients and is commonly used in clinical trials [7].

Distribution and anchor derivations often yielded different values, which may be partly attributed to varying patient population characteristics and follow-up length of time across studies. MCID anchor-derived values for KOOS, JR were greater than those obtained by distribution method [22, 24], the latter being also observed to not exceed distribution-derived MDC values [24]. One such reason may be the lack of consistency of anchor scales, the anchors chosen themselves, and subsequent dependence on patient interpretation. Anchor scales and the specific anchor on which clinical improvement of significance is defined are arbitrarily chosen. Scales that are more nuanced (e.g. a quantitative 10-point Likert scale) can detect incremental change that may translate to clinical significance earlier compared to scales with a larger range between data points. Scales with a larger range between data points (e.g. none, very mild, mild, moderate, and great improvement) may require the patient to experience a dramatic change for clinically significant change to be reported. Baseline scores may impact patient assessment of improvement as well. For example, Tubach et al. reported MCII values varied depending on baseline visual analog scale pain scores. Patients with severe pain required a higher level of change to consider themselves clinically improved [40]. Additionally, the one anchor question posed often varies across studies and may not be validated nor wholly representative of the true breadth of change associated with the intervention. Lastly, the heterogeneity of anchor derivation methods, ranging from ROC-curve analysis to simple linear regression, also contributes to the lack of consistency.

Distribution methods result in MDC values that describe statistical significance and do not capture clinical change as directly perceived by the patient. MDC values can only be taken with a degree of certainty that any change beyond that merely associated with the variability of repeated PROM measurements is truly significant. Since these values are based on the SEM and PROM reliability, they are not interchangeable with MCID or other anchor-derived clinical improvement values. The SEM includes the SD for a given population and thus, may not be widely generalizable. Furthermore, its basis on the SD leaves MDC derivations susceptible to sample size.

Patient factors such as age, gender, and BMI can be predictors of outcomes, which has implications for patient selection preoperatively. Specifically, PASS thresholds have been shown to be higher in men compared to women and in those with higher preoperative SF-36 physical and mental scores (> 50), suggesting greater change is necessary for the achievement of an acceptable symptom state in certain subgroups [9]. The identification of patient factors that may affect the attainment of a postoperative satisfaction threshold has implications for patient selection.

As the repayment structure moves toward a performance value-based system, standardization and consistent use of clinical improvement metrics determining efficacy become increasingly critical. For example, the Center for Medicare and Medicaid Services (CMS) has recently funded the development of guidelines to advise developers on patient-reported outcome performance measures (PRO-PM) for use in CMS-funded value-based purchasing programs. This highlights the timeliness in which the performance measurement landscape is evolving to ultimately improve quality and reduce costs. PROM interpretability, among others, is one example of a quality measure examined by CMS to develop standardized measures goals for achieving high-value care [41]. Current PRO-PMs in the CMS measures inventory tool include KOOS, KOOS, JR, PROMIS-10 Global Health, and Veterans RAND-12 for functional status assessment after TKA [42].

We recommend future research should focus on more clearly delineated definitions of clinical change to establish consistency across studies and avoid misuse and misinterpretation of terms among researchers and clinicians. There should be consensus on methods of calculation and anchor questions employed. Greater standardization of clinical improvement reporting will have implications for patient stratification preoperatively and appropriateness of surgical intervention, ultimately improving patient satisfaction and outcomes.

## Conclusion

There is low standardization of metrics of clinical significance across a variety of PROMs and methods of derivation in TKA literature. Consistent interpretation and application of PROMs following TKA in both clinical and research settings necessitate the standardization of methods used to obtain clinical significance values to ultimately improve quality and patient satisfaction.

## Supplementary Information

**Additional file 1.** Modes of Calculation in the Literature.

## Data Availability

The datasets used and/or analyzed during the current study are available from the corresponding author upon reasonable request.

## Abbreviations

PROMs: Patient-reported outcome measures
TKA: Total knee arthroplasty
MCID: Minimal clinically important difference
CID: Clinically important difference
MCII: Minimal clinically important improvement
MDC: Minimal detectable change
SCB: Substantial clinical benefit
MID: Minimal important difference
MIC: Minimal important change
PASS: Patient acceptable symptom state
THA: Total hip arthroplasty
SDD: Smallest detectable difference
MCIC: Minimal clinically important change
PRISMA: Preferred Reporting Items for Systematic Review and Meta-Analysis
KOOS: Knee Injury and Osteoarthritis Outcome Score
WOMAC: Western Ontario McMaster University osteoarthritis index
SF-12: Short Form-12
SF-36: Short Form-36
OKS: Oxford Knee Score
PROMIS: Patient-Reported Outcomes Measurement Information System
ICOAP: Intermittent and Constant Osteoarthritis Pain
EQ-5D-3L: EuroQoL 5-dimension 3-level
VAS: Visual Analog Scale
NRS: Numeric Rating Scale
CAT: Computerized adaptive test
QOL: Quality of life
SAPS: Self-Administered Patient Satisfaction Scale
HSS: Hospital for Special Surgery
SD: Standard deviation
PCS: Physical component scores
MCS: Mental component scores
SEM: Standard error of measurement
ROC: Receiver operating characteristic curves
KSKS: Knee Society Knee Score
KSFS: Knee Society Function Score
BMI: Body mass index
CMS: Center for Medicare and Medicaid Services
PRO-PM: Patient-reported outcome performance measures

## References

[1] Rolfson O, Bohm E, Franklin P, Lyman S, Denissen G, Dawson J (2016). Patient-reported outcome measures in arthroplasty registries. Acta Orthop.

[2] Ramkumar PN, Harris JD, Noble PC (2015). Patient-reported outcome measures after total knee arthroplasty. Bone Jt Res.

[3] Jaeschke R, Singer J, Guyatt GH (1989). Measurement of health status: ascertaining the minimal clinically important difference. Control Clin Trials.

[4] Hays RD, Woolley JM (2000). The concept of clinically meaningful difference in health-related quality- how Meaningful is it?. Pharmacoeconomics.

[5] Chesworth BM, Mahomed NN, Bourne RB, Davis AM (2008). Willingness to go through surgery again validated the WOMAC clinically important difference from THR/TKR surgery. J Clin Epidemiol.

[6] Sayers A, Wylde V, Lenguerrand E, Gooberman-Hill R, Dawson J, Beard D (2017). A unified multi-level model approach to assessing patient responsiveness including; Return to normal, minimally important differences and minimal clinically important improvement for patient reported outcome measures. BMJ Open.

[7] Beard DJ, Harris K, Dawson J, Doll H, Murray DW, Carr AJ (2015). Meaningful changes for the Oxford hip and knee scores after joint replacement surgery. J Clin Epidemiol.

[8] Glassman SD, Copay AG, Berven SH, Polly DW, Subach BR, Carreon LY (2008). Defining substantial clinical benefit following lumbar spine arthrodesis. J Bone Joint Surg Am.

[9] Kunze KN, Fontana MA, Maclean CH, Lyman S, Mclawhorn AS (2022). Defining the patient acceptable symptom State. J Bone Joint Surg Am.

[10] Moher D, Liberati A, Tetzlaff J, Altman DG, Altman D, Antes G, et al. Preferred reporting items for systematic reviews and meta-analyses: The PRISMA statement. PLoS Med. 2009;6. 10.1371/journal.pmed.1000097. PMC3090117 21603045

[11] Berliner JL, Ba DJB, Mph VC, Soohoo NF, Bozic KJ (2017). Can Preoperative patient-reported outcome measures be used to predict meaningful improvement in function after TKA ?. Clin Orthop Relat Res.

[12] Blevins JL, Chiu YF, Lyman S, Goodman SM, Mandl LA, Sculco PK (2019). Comparison of expectations and outcomes in rheumatoid arthritis versus osteoarthritis patients undergoing total knee arthroplasty. J Arthroplasty.

[13] Busija L, Osborne RH, Nilsdotter A, Buchbinder R, Roos EM (2008). Magnitude and meaningfulness of change in SF-36 scores in four types of orthopedic surgery. Health Qual Life Outcomes.

[14] Clement ND, MacDonald D, Simpson AHRW (2013). The minimal clinically important difference in the Oxford knee score and Short Form 12 score after total knee arthroplasty. Knee Surg Sport Traumatol Arthrosc.

[15] Clement ND, Bardgett M, Weir D, Holland J, Gerrand C, Deehan DJ (2018). What is the Minimum Clinically Important Difference for the WOMAC Index After TKA?. Clin Orthop Relat Res.

[16] Clement ND, Weir D, Holland J, Gerrand C, Deehan DJ (2019). Meaningful changes in the Short Form 12 physical and mental summary scores after total knee arthroplasty. Knee.

[17] Connelly JW, Galea VP, Rojanasopondist P, Matuszak SJ, Ingelsrud LH, Nielsen CS (2019). Patient acceptable symptom State at 1 and 3 years after total Knee Arthroplasty: Thresholds for the Knee Injury and Osteoarthritis Outcome Score (KOOS). J Bone Jt Surg Am.

[18] Fontana MA, Lyman S, Sarker GK, Padgett DE, MacLean CH (2019). Can machine learning algorithms predict which patients will achieve minimally clinically important differences from total joint arthroplasty?. Clin Orthop Relat Res.

[19] Goodman SM, Mehta B, Mandl LA, Szymonifka J, Finik J, Figgie M (2020). Validation of the Hip Disability and Knee Injury and Osteoarthritis Outcome Score (HOOS, KOOS) pain and function subscales for use in Total Hip (THR) and Total Knee Replacement (TKR) clinical trials. J Arthroplasty.

[20] Harris AHS, Kuo AC, Bowe TR, Manfredi L, Lalani NF, Giori NJ (2021). Can Machine learning methods produce accurate and easy-to-use preoperative prediction models of one-year improvements in pain and functioning after knee arthroplasty?. J Arthroplasty.

[21] Kagan R, Anderson MB, Christensen JC, Peters CL, Gililland JM, Pelt CE (2018). The recovery curve for the patient-reported outcomes measurement information system patient-reported physical function and pain interference computerized adaptive tests after primary total knee arthroplasty. J Arthroplasty.

[22] Kuo AC, Giori NJ, Bowe TR, Manfredi L, Lalani NF, Nordin DA (2020). Comparing methods to determine the minimal clinically important differences in patient-reported outcome measures for veterans undergoing elective total hip or knee arthroplasty in veterans health administration hospitals. JAMA Surg.

[23] Lawrie CM, Abu-Amer WY, Clohisy JC (2021). Is the patient-reported outcome measurement information system feasible in bundled payment for care improvement total knee arthroplasty patients?. J Arthroplasty.

[24] Lyman S, Lee Y-Y, McLawhorn AS, Islam W, MacLean CH (2018). What are the minimal and substantial improvements in the HOOS and KOOS and JR versions after total joint replacement?. Clin Orthop Relat Res.

[25] Maxwell J, Niu J, Singh JA, Nevitt MC, Law LF, Felson D (2013). The Influence of the contralateral knee prior to knee arthroplasty on post-arthroplasty function: the multicenter osteoarthritis Study. J Bone Joint Surg Am.

[26] Razak HRBA, Tan CS, Chen YJD, Pang HN, Darren Tay KJ, Chin PL (2016). Age and preoperative knee society score are significant predictors of outcomes among asians following total knee arthroplasty. J Bone Jt Surg Am.

[27] Peer MA, Lane J (2013). The knee injury and osteoarthritis outcome score (KOOS): a review of its psychometric properties in people undergoing total knee arthroplasty. J Orthop Sports Phys Ther.

[28] Roos EM, Roos HP, Lohmander LS, Ekdahl C, Beynnon BD (1998). Knee Injury and Osteoarthritis Outcome Score (KOOS) - Development of a self-administered outcome measure. J Orthop Sports Phys Ther.

[29] Bellamy N, Buchanan WW, Goldsmith CH, Campbell JSL (1988). Validation study of WOMAC: a health status instrument for measuring clinically important patient relevant outcomes to antirheumatic drug therapy in patients with osteoarthritis of the hip or knee. J Rheumatol.

[30] Ware J, Kosinski M, Keller SD. A 12-Item Short-Form Health Survey : Construction of Scales and Preliminary Tests of Reliability and Validity Author ( s ): John E . Ware , Jr ., Mark Kosinski and Susan D . Keller Published by : Lippincott Williams & Wilkins Stable URL : http://www.jstor. Med Care. 1996;34:220–33. 10.1097/00005650-199603000-00003 8628042

[31] Laucis NC, Hays RD, Bhattacharyya T (2014). Scoring the SF-36 in orthopaedics: a brief guide. J Bone Jt Surg Am.

[32] J.E. Ware CDS. The MOS 36-Item Short-Form Health Survey (SF-36): I . Conceptual Framework and Item Selection Author (s): John E . Ware , Jr . and Cathy Donald Sherbourne Published by : Lippincott Williams & Wilkins Stable URL : http://www.jstor.org/stable/3765916. Ac. Med Care 1992;30:473–83. 1593914

[33] Whitehouse SL, Blom AW, Taylor AH, Pattison GTR, Bannister GC (2005). The Oxford Knee Score; problems and pit falls. Knee.

[34] Dawson J, Fitzpatrick R, Murray DCA (1998). Questionnaire on the perceptions of patients about total knee replacement. J Bone Jt Surg Br.

[35] Brodke DJ, Saltzman CL, Brodke DS (2016). PROMIS for orthopaedic outcomes measurement. J Am Acad Orthop Surg.

[36] Hawker GA, Davis AM, French MR, Cibere J, Jordan JM, March L (2008). Development and preliminary psychometric testing of a new OA pain measure - an OARSI/OMERACT initiative. Osteoarthr Cartil.

[37] Parkin DW, Do Rego B, Shaw R (2022). EQ-5D-3L and quality of life in total knee arthroplasty (TKA) patients: beyond the index scores. J Patient Rep Outcomes.

[38] Noiseux NO, Callaghan JJ, Clark CR, Zimmerman MB, Sluka KA, Rakel BA (2014). Preoperative predictors of pain following total knee arthroplasty. J Arthroplasty.

[39] Naylor JM, Hayen A, Davidson E, Hackett D, Harris IA, Kamalasena G (2014). Minimal detectable change for mobility and patient-reported tools in people with osteoarthritis awaiting arthroplasty. BMC Musculoskelet Disord.

[40] Tubach F, Ravaud P, Baron G, Falissard B, Logeart I, Bellamy N (2005). Minimal clinically important improvement. Ann Rheum Dis.

[41] National Quality Forum (2013). Patient Reported Outcomes (PROs) in Performance Measurement.

[42] Measures C for M and MS, Tool I. Functional Status Assessment for Total Knee Replacement. Centers Medicare Medicaid Meas Invent Tool. 2022. https://cmit.cms.gov/cmit/#/MeasureView?variantId=4834&sectionNumber=1. Accessed 16 Nov 2022.

