# Supplementary material for: Defining a successful total knee arthroplasty: a systematic review of metrics of clinically important changes
Source: Arthroplasty. 2023 May 18;5:25. doi: 10.1186/s42836-023-00178-3 (PMC10193600; doi:10.1186/s42836-023-00178-3)
Supplement: Supplementary file 1 — Additional file 1. Modes of Calculation in the Literature. [file 42836_2023_178_MOESM1_ESM.docx]

**Additional File 1**

**Modes of Calculation in the Literature**

Anchor-based methods apply a subjective clinical question (e.g. “Would you go through this surgery again?”) or global assessment rating scale to the change in PROM scores to determine their significance. For example, a PROM score numerical change corresponding to “a great deal better” on the anchor scale, typically a variation of an X-point Likert scale, would constitute the MCID. Anchor-based values were obtained using simple linear regression analysis [14] and/or receiver operating characteristic curves (ROC) at maximum sensitivity and specificity [5,15,17, 22,24].

ROC curves identify PROM change scores that generally distinguish between those who are “better” from unchanged (or those willing versus unwilling to undergo surgery again) using anchor responses. The degree of sensitivity indicates the extent to which patients that have reported improvement actually have PROM change scores that meet or are above the MCID. The degree of specificity indicates the extent to which patients who have reported no improvement have PROM change scores that are below the MCID. The area under the curve (AUC) indicates the probability that these change scores correctly distinguish between these groups, with an AUC above 0.70 considered acceptable [Copay].

For PASS specifically, a combination of anchor methods was used: 80% specificity using ROC curves, the Youden index (maximum sensitivity and specificity), and the 75^th^ percentile. The latter method refers to 75 percent of patients reporting improvement with change scores greater than the PASS threshold [17].

Distribution methods compare PROM change scores to errors of measurement. The standard error of measurement (SEM) is the error associated with the measurement tool below which any change in scores is attributed to measurement error rather than true change. SEM is calculated using the standard deviation (SD) and reliability of the measurement tool (SEM = SD x √1-reliability), where the reliability of a PROM and its components is calculated using Cronbach’s alpha. Cronbach’s alpha measures the internal consistency of a given scale [Tavakol]. The MDC is the minimum change detected that is above the measurement error reported within a certain degree of confidence. A 95% CI indicates a 95% chance the patient’s true score lies within the reported range. MDC-95 is obtained by multiplying the SEM by the respective z-score and √2 [15,39].

Distribution methods also involve the standard deviation. One-half the standard deviation among a group of scores has been previously published as one method to obtain MCID values and is known to carry universality across many studies and applications [Norman].
